# Efficacy, Safety and Tolerability of Volixibat, an IBAT Inhibitor, in Patients With Intrahepatic Cholestasis of Pregnancy

**DOI:** 10.1111/liv.70523

**Published:** 2026-01-28

**Authors:** Caroline Ovadia, Sophia Stone, Baha Sibai, Tiago Nunes, Douglas B. Mogul, Jayshree Krishnaswami, Jennifer Kahng, Furong Li, Qurratul Ann Warsi, Elaine Chien, Pamela Vig, Catherine Williamson

**Affiliations:** ^1^ Department of Women and Children's Health King's College London London UK; ^2^ Centre for Reproductive Health, Institute for Regeneration and Repair University of Edinburgh Edinburgh UK; ^3^ St Richard's Hospital University Hospitals Sussex NHS Foundation Trust Chichester UK; ^4^ Division of Maternal‐Fetal Medicine, Department of Obstetrics, Gynecology and Reproductive Sciences, McGovern Medical School, Texas Medical Center The University of Texas Houston Texas USA; ^5^ Mirum Pharmaceuticals, Inc. Foster City California USA; ^6^ Institute of Reproductive and Developmental Biology Imperial College London London UK

**Keywords:** bile acids and salts, cholestasis, pregnancy outcome, pruritus

## Abstract

Intrahepatic cholestasis of pregnancy (ICP) presents with cholestatic pruritus, elevated sBA and increased risk of adverse perinatal outcomes. Volixibat is a minimally absorbed IBAT inhibitor that interrupts enterohepatic recirculation. We describe four patients with ICP with pruritus and sBA > ULN treated with volixibat (20 or 80 mg BID orally until delivery), with dose modifications permitted for tolerability. Daily pruritus scores, sBA, liver enzymes, perinatal outcomes and TEAEs were assessed. Over 1000 patients were invited to participate; 26 were screened, and four received volixibat. Three patients experienced reductions in pruritus from baseline regardless of volixibat dose, with intermittent relief coinciding with resumption of dosing when doses were interrupted. sBA nadir values all reached < 6 μmol/L following volixibat. No clinically meaningful changes in laboratory parameters were observed. All patients had healthy live births. Most frequent TEAEs were gastrointestinal. Volixibat demonstrated improvements in pruritus and sBA in patients with ICP.

**Trial Registration:** NCT04718961

## Key Points

1. Volixibat treatment for ICP resulted in reduced serum bile acid concentrations and improvement in pruritus in the majority of study participants.
2. The main adverse event was diarrhoea that improved with reduction of volixibat dose.
3. No drug was detected in maternal blood.

**Abbreviations**
AE: adverse event
ALGS: Alagille syndrome
BID: twice daily
IBAT: ileal bile acid transporter
ICP: intrahepatic cholestasis of pregnancy
ItchRO: Itch‐Reported Outcome
PBC: primary biliary cholangitis
PFIC: progressive familial intrahepatic cholestasis
PSC: primary sclerosing cholangitis
sBA: serum bile acid
TEAE: treatment‐emergent adverse event
UDCA: ursodeoxycholic acid

## 1 Introduction

Intrahepatic cholestasis of pregnancy (ICP) is the most common liver disorder of pregnancy. Typically, ICP affects 0.7% of pregnant women of European ancestry; it is twice as common in women of South Asian ethnicity [1] and affects approximately 3% of women from the Andean regions of Latin America. Presenting features of ICP are maternal pruritus, raised serum bile acid (sBA) concentrations and hepatic impairment [2]. The pruritus is of variable severity but can prevent sleep and impact the mood of affected women. Complications of ICP can include adverse pregnancy outcomes (e.g., preterm birth, meconium‐stained amniotic fluid, intrauterine fetal death and prolonged admission to the neonatal unit) [3, 4]. The extent of maternal hypercholanemia is related to the risk of some pregnancy complications; in women with sBA concentrations of ≥ 40 μmol/L, there is a significantly increased risk of spontaneous preterm birth and meconium‐stained amniotic fluid, and when concentrations are ≥ 100 μmol/L, there is an increased risk of stillbirth, particularly from 35 weeks' gestation [4, 5].

The medication most commonly used to treat ICP is ursodeoxycholic acid (UDCA). While UDCA has limited impact on pruritus severity [6], it has been shown to reduce the rate of spontaneous preterm birth in ICP [7]. Treatment with UDCA reduces serum concentrations of liver transaminases and the chance of meconium‐stained amniotic fluid, but randomised placebo‐controlled trials have given mixed results regarding the impact on sBA concentrations [6].

Ileal bile acid transporter (IBAT) inhibitors prevent uptake of bile acids from the gut lumen by the ileal apical sodium‐dependent bile acid cotransporter (also called ASBT/*SLC10A2*). They are effective at reducing hypercholanemia and pruritus in adult and paediatric cholestatic disorders. Specifically, the IBAT inhibitor maralixibat has demonstrated not only statistically significant and clinically meaningful reductions in pruritus, sBA levels and bilirubin but also improvements in quality of life across several cholestatic disorders, including progressive familial intrahepatic cholestasis (PFIC), Alagille syndrome (ALGS), primary sclerosing cholangitis (PSC) and primary biliary cholangitis (PBC) [8, 9, 10, 11]. The impact of IBAT inhibitors on children with PFIC associated with *ABCB11* and *ABCB4* variants is particularly relevant to ICP, as approximately 20% of affected women have heterozygous pathogenic variants in these genes [12, 13].

The aim of the OHANA trial was to evaluate the safety, tolerability and efficacy of the IBAT inhibitor volixibat when used to treat ICP in women with elevated sBA concentrations and symptomatic pruritus.

## 2 Methods

### 2.1 Trial Design and Participants

OHANA (NCT04718961) was a phase 2a/2b, randomised, double‐blinded, placebo‐controlled adaptive clinical trial consisting of two parts (Figure S1). Part 1 was a proof‐of‐concept open‐label study that assessed volixibat dosing range. The goal of Part 2 (double‐blinded randomised period) was to compare the volixibat dose selected from Part 1 with placebo to determine superior efficacy. Because of the early termination of the OHANA trial due to lack of enrollment, data were analysed for the four participants enrolled during Part 1 of the trial and are summarised in this report. Key eligibility criteria and additional methodology are included in the Supporting Information.

### 2.2 Treatment

After a screening period of up to 10 days, participants were randomised to either 20 or 80 mg twice daily (BID) of volixibat. Study medication dosing began on Day 0 and continued until the end of the treatment period, defined as the day of birth. Dose reduction was permitted at the clinician's discretion. As much as possible, each dose of study medication was administered approximately 30 min before meals. The first dose of study medication was administered under observation in the clinic; monitoring of fetal well‐being was performed before and after dosing.

### 2.3 Assessments

Efficacy of volixibat on pruritus was assessed by change from baseline to Week 3 of the treatment period (or at birth if this occurred < 3 weeks after commencement of volixibat) in weekly average worst daily itch score as measured by adult Itch‐Reported Outcome (ItchRO), an 11‐point numeric rating scale of worst itch severity ranging from 0 (no itch) to 10 (worst possible itch).

The safety and tolerability of volixibat in participants with ICP during Part 1 were assessed based on the following endpoints: (1) proportion of participants experiencing one or more treatment‐emergent adverse events (TEAEs), serious adverse events (AEs), AEs of special interest, events of clinical interest, or AEs leading to discontinuation of study medication; and (2) proportion of participants experiencing one or more clinically significant laboratory abnormalities. Safety assessments included AEs, clinical laboratory tests, vital signs and physical examinations. Additional fetal safety assessments, including serial ultrasound scans to assess fetal growth and nonstress test/cardiotocography or fetal biophysical profile, were also completed.

Assessments for exploratory objectives included (1) volixibat drug levels in maternal and fetal serum and pharmacodynamic markers in maternal blood in participants with ICP and (2) the effect of volixibat on additional perinatal outcomes.

### 2.4 Data and Statistical Analyses

The enrolled population consisted of all participants who signed an informed consent form and successfully completed screening. The safety cohort consisted of all participants who received one or more doses of study medication and were classified based on treatment received.

For all safety and tolerability analyses, participants were analysed by the treatment dose received. Since only four participants completed the trial, summary statistics were not calculated. Instead, by‐participant listings were provided for all safety and tolerability variables.

## 3 Results

### 3.1 Patient Disposition and Baseline Demographics

**FIGURE 1:**
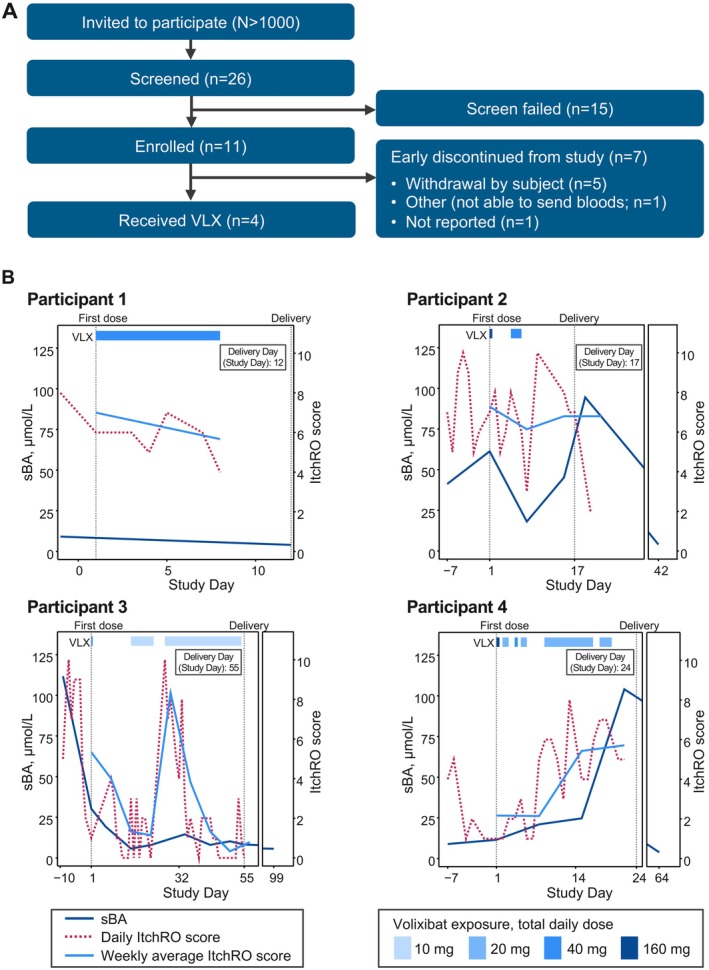
(A) Participant disposition. The schematic depicts actual enrollment completed for the OHANA trial. (B) Changes in sBA levels, daily ItchRO scores and weekly average ItchRO scores in the trial participants 1–4. The figure depicts results for each of the three endpoints following initiation and discontinuation of volixibat treatment in enrolled participants. ItchRO, Itch‐Reported Outcome; sBA, serum bile acid; VLX, volixibat.

OHANA comprised 25 sites in three countries (New Zealand, United Kingdom and United States) and was conducted from May 25, 2021, through December 7, 2022. The study was terminated by the sponsor in November 2022 due to challenges in enrollment in this high‐risk pregnancy population (Figure 1A). Key demographic features of the four participants who received volixibat are shown in Table 1.

**TABLE 1:** Key baseline demographics and associated maternal and perinatal outcomes.

Participant	1	2	3	4
Key baseline demographics				
Age, years	20	25	25	38
Pruritus, ItchRO^a^	7.0	7.3	5.3	2.2
Peak sBA level prior to first dose, μmol/L	13	43	112	12
Initial dose of VLX, mg	40	160	40	160
Treatment duration, wk	1.1	0.6	5.3	2.6
Prior and/or concomitant UDCA usage, dose	No	No	Yes, 500 mg BID^b^	Yes, 500 mg BID^c^
Maternal and perinatal outcomes				
Pruritus, change from baseline to end of treatment, ItchRO	−1.3	−2.0	−4.5	3.5
sBA, change from baseline to end of treatment, μmol/L	−4.2	51.5^d^	−22	−6.6
Gestational age at delivery, wk/d	37/6	35/4	37/1	34/4
Delivery type/mode	Full/V	Preterm/V	Full/C	Preterm/C
Labor type	Iatrogenic related to ICP	Spontaneous	Iatrogenic related to ICP	Iatrogenic related to ICP
Live birth vs. stillbirth	Live	Live	Live	Live
NNU admission	No	Yes	No	Yes (respiratory support needed)
Maternal complications	Yes^e^	No	No	No
Postpartum haemorrhage	No	No	No	No
ALT change from baseline to end of treatment, U/L	2	4	44^f^	17
VLX concentrations in maternal venous blood, ng/mL	< 0.05	< 0.05	< 0.05	< 0.05
VLX concentrations in umbilical venous blood, ng/mL	NA	NA	< 0.05	NA

Abbreviations: ALT, alanine aminotransferase; BID, twice daily; C, caesarean; ICP, intrahepatic cholestasis of pregnancy; ItchRO, Itch‐Reported Outcome; NA, not available; NNU, neonatal unit; QD, once daily; sBA, serum bile acid; UDCA, ursodeoxycholic acid; V, vaginal; VLX, volixibat.

^a^ Baseline pruritus is the average weekly ItchRO score prior to volixibat treatment.

^b^ Participant took UDCA prior to and during trial participation (Days −6 to 54).

^c^ Participant took UDCA during the trial (Days 15 to 22) but not prior due to elevated sBA levels. Participant received additional dosages on Days 22 to 23 (500 mg QD) and Days 22 to 25 (1 g BID).

^d^ Upon follow‐up 3 weeks later, sBA change from baseline was −38.1 μmol/L.

^e^ Preeclampsia with severe features.

^f^ Upon follow‐up 6 weeks later, ALT change from baseline was −129 U/L.

### 3.2 Efficacy

Changes in sBA levels, daily ItchRO scores and weekly average ItchRO scores for each of the four participants over the course of the trial are shown in Figure 1B, and additional maternal and perinatal outcomes are listed in Table 1. Following treatment with volixibat, all participants experienced a reduction of sBA levels, with sustained reductions observed over time in three participants (Figure 1B). For three participants, there was a reduction in sBA concentrations at the time of taking volixibat. All sBA nadir values reached < 6 μmol/L following volixibat treatment, either by the delivery date or during follow‐up assessments. Three participants experienced reductions in pruritus, with marked improvements in pruritus scores regardless of volixibat dose and intermittent relief coinciding with the resumption of volixibat dosing when treatment had been interrupted. No significant deficiencies were observed in vitamin A, D and E levels for all participants.

None of the participants had a stillbirth, two delivered at full term and two delivered preterm (Table 1). Both preterm newborns required neonatal unit admission. One participant experienced additional pregnancy‐related complications unrelated to volixibat treatment (preeclampsia with severe features).

### 3.3 Safety

No clinically meaningful changes in liver enzyme levels or hematology parameters were observed following treatment with volixibat (Table 1). Serum volixibat levels were minimally detected (< 0.05 ng/mL) in maternal (*n* = 4) and umbilical venous (*n* = 1) blood.

The most frequently reported TEAEs were gastrointestinal (Table S1). Three of four participants experienced diarrhoea and/or abdominal cramping leading to dose reduction, treatment interruption and/or early discontinuation. One participant tolerated treatment until delivery with no dose modifications due to AEs. At the end of treatment, volixibat concentrations in maternal and umbilical venous blood were < 0.05 ng/mL. None of the TEAEs triggered premature labor.

## 4 Conclusions

This report of four women with ICP treated with volixibat showed an improvement in pruritus and a sustained reduction in sBA. The principal maternal AE was diarrhoea, which was severe in two participants, both of whom received the higher volixibat dose. With a reduction in volixibat dose, the symptoms of diarrhoea improved in both women. The reduction in sBA concentrations observed following volixibat treatment is of clinical importance due to the concern about spontaneous preterm birth and stillbirth in ICP cases with high sBA concentrations [4, 5]. Three study participants had sBA concentrations above 40 μmol/L, and volixibat treatment reduced sBA levels in two. No significant deficiencies in fat‐soluble vitamins were observed following volixibat treatment; however, vitamin K was not collected.

The reductions in sBA concentrations and severity of pruritus are consistent with the impact of maralixibat, another IBAT inhibitor, on these cholestasis‐associated disease features in nonpregnant individuals in PSC, PBC, ALGS and PFIC [8, 9, 10, 11, 14, 15]. The positive results from the trial in patients with PFIC, which included the largest number of PFIC types ever studied, are of potential relevance to women with ICP, as pathogenic heterozygous nontruncating variants in *ABCB11* or susceptibility loci that increase the risk of ICP are reported in ICP cases [12, 13, 16, 17].

In the current study, AEs occurred more commonly in those taking higher doses of volixibat, similar to what has been reported for other IBAT inhibitors [14, 15]. Importantly, the participants in OHANA who received lower volixibat doses did not have diarrhoea that was sufficiently severe to necessitate cessation of treatment. Previous studies of volixibat did not indicate that the high dose used in the OHANA trial would result in marked diarrhoea [18]. Alteration of volixibat dose did not influence the frequency or severity of these AEs, while in the current trial, a reduction in dose did appear to reduce the frequency and severity of diarrhoea in those who initially received the higher dose of the medication. Thus, future studies of IBAT inhibitors in ICP should consider starting treatment at lower doses with gradual dose increases. The low concentration of volixibat in maternal serum is consistent with previous reports that it is minimally absorbed, likely indicating that the fetus will have limited exposure to the medication [18, 19].

There are several limitations in this trial mainly due to sample size. Since only four participants were enrolled in the trial, formal statistical analyses could not be conducted, and only descriptive statistics and individual data for each participant were reported. While there was no control group in this trial due to the fact that this was a proof of concept, dose finding study, outcomes reported here with volixibat were similar to those reported with maralixibat in ICP [20]. Although OHANA was originally planned as a larger scale clinical trial, since only four participants were enrolled, these results reflect what is typically reported in a case series. While the efficacy and tolerability data with volixibat in ICP are promising, these results should be considered hypothesis generating. This study provided early signals of volixibat's potential as a treatment in ICP, but further research needs to be conducted in order to adequately determine the impact of the medication.

In summary, our study shows the potential utility of IBAT inhibitors for the management of cholestatic pregnancies, demonstrating improvements in pruritus and sBA levels. With safety data indicating no adverse fetal event and minimal to no fetal exposure to the medication, this is a promising new avenue of treatment. Patients with ICP appear more susceptible to the gastrointestinal side effects of the medication than those with chronic cholestatic disease; hence, future strategies may necessitate gradual dose increases, titrating to symptoms. Nevertheless, given the absence of any effective alternative treatment for ICP beyond premature delivery, the potential value of this as a novel treatment warrants further research.

## Funding

The study was funded by Mirum Pharmaceuticals Inc.

## Ethics Statement

The trial complied with consensus ethical principles derived from international guidelines, including the Declaration of Helsinki and the Council for International Organisations of Medical Sciences International Ethical Guidelines, applicable International Council for Harmonisation of Technical Requirements for Pharmaceuticals for Human Use, Good Clinical Practice guidelines and applicable laws and regulations. The trial protocol, protocol amendments and informed consent documents were approved by an institutional review board or independent ethics committee at each site.

## Consent

Informed written consent was obtained from all patients for being included in the study.

## Conflicts of Interest

Caroline Ovadia is a consultant for Mirum Pharmaceuticals Inc. Sophia Stone and Baha Sibai have nothing to disclose. Tiago Nunes is an employee of and shareholder in Mirum Pharmaceuticals Inc. Douglas B. Mogul is an employee of and shareholder in Mirum Pharmaceuticals Inc. Jayshree Krishnaswami is an employee of and shareholder in Mirum Pharmaceuticals Inc. Jennifer Kahng is an employee of and shareholder in Mirum Pharmaceuticals Inc. Furong Li is a former employee of and shareholder in Mirum Pharmaceuticals Inc. Qurratul Ann Warsi is a former employee of and shareholder in Mirum Pharmaceuticals Inc. Elaine Chien is a former employee of and shareholder in Mirum Pharmaceuticals Inc. Pamela Vig is an employee of and shareholder in Mirum Pharmaceuticals Inc. Catherine Williamson is a paid consultant for Mirum Pharmaceuticals Inc., and Ipsen; previously advised GSK, and was paid to deliver a webinar by Advanz Pharma and a lecture by Ipsen.

## Supporting information


**Figure S1:** Study Design: Open‐Label Proof‐of‐Concept Phase for OHANA Trial.
**Table S1:** Safety Outcomes.

## Data Availability

Beginning 6 months and ending 5 years after publication, deidentified participant data for data meta‐analysis might be made available to investigators whose proposed use of the data has been approved by a review committee, including the primary authors and the study funder. The study protocol will also be available via web link. Proposals should be directed to grants@mirumpharma.com Before being granted access, data requesters will be required to sign a data access agreement.
