# Supplementary material for: Efficacy, Safety and Tolerability of Volixibat, an IBAT Inhibitor, in Patients With Intrahepatic Cholestasis of Pregnancy
Source: Liver Int. 2026 Jan 28;46(3):e70523. doi: 10.1111/liv.70523 (PMC12849984; doi:10.1111/liv.70523)
Supplement: Supplementary file 1 — Figure S1: Study Design: Open‐Label Proof‐of‐Concept Phase for OHANA Trial. Table S1: Safety Outcomes. [file LIV-46-0-s001.pdf]

Efficacy, Safety, and Tolerability of Volixibat, an IBAT Inhibitor, in Patients With Intrahepatic Cholestasis of Pregnancy

Caroline Ovadia, et al.

SUPPORTING INFORMATION

SUPPORTING METHODS

The OHANA (NCT04718961) trial assessed the efficacy, safety, and tolerability of volixibat in adult women with intrahepatic cholestasis of pregnancy (ICP) and elevated serum bile acid (sBA) concentrations and consisted of two parts. A prespecified interim analysis was planned at the end of Part 1 to assess safety and tolerability, pharmacokinetics, and dose exploration based on efficacy and tolerability.

Eligibility Criteria

Key eligibility criteria were female biological sex, age between ≥18 and ≤45 years with viable pregnancy of 20 weeks 0 days or above, singleton gestation of no more than 37 weeks 0 days (inclusive) or twin gestation of no more than 35 weeks 0 days (inclusive) at the baseline visit (Day 0), onset of pruritus during pregnancy with no known etiology other than ICP, documented sBA level ≥10 μmol/L at any point during the current pregnancy, and absence of known pathology that may produce similar laboratory findings or symptoms (eg, primary biliary cholangitis, primary sclerosing cholangitis). Key exclusion criteria included scheduled delivery within seven days of screening or baseline visit; presence of triplets or higher multifetal gestation; known placenta accreta;

complete placenta previa; premature rupture of membranes at any gestational age prior to randomization; cervical insufficiency; history of prior spontaneous birth at ≤34 weeks not secondary to known or suspected ICP or other condition likely to result in spontaneous or iatrogenic delivery before 37 weeks; known nonreassuring fetal status based upon antepartum testing; and evidence of current underlying cholestatic liver disease other than ICP, other hepatobiliary conditions, known or suspected inflammatory bowel disease, history of small bowel resection or bariatric surgery, or concurrent underlying condition with unstable pruritus at screening or baseline.

Treatment and Assessments

Monitoring of fetal well-being was performed before and after dosing via nonstress tests (NSTs)/cardiotocography (CTG), fetal biophysical profile (BPP), or auscultation/visualization of fetal cardiac activity if NST/CTG and/or BPP were not feasible due to gestational age.

Adult Itch-Reported Outcome was rated over the past 24 hours and completed daily in an eDiary within a prespecified time window. Evaluation of the impact of volixibat on a composite perinatal outcome in participants with ICP (proportion of participants experiencing one or more perinatal deaths, spontaneous preterm birth, iatrogenic preterm birth attributable to ICP or ICP-related complications, or neonatal unit admission for ≥12 hours from birth until hospital discharge).

An adverse event (AE) was defined as any untoward medical occurrence in a clinical study participant or the newborn temporally associated with the use of volixibat, regardless of whether it was considered related to the study medication. This could

include the following potential events: any abnormal laboratory test results or other safety assessments, including those that worsen from baseline and are considered clinically significant in the medical and scientific judgement of the investigator. The severity of the AE was assessed by the investigator according to the National Cancer Institute Common Terminology Criteria for Adverse Events (CTCAE), version 5.0 or higher. If an AE was not listed in the CTCAE, the following grading system was used: grade 1 (mild), grade 2 (moderate), grade 3 (severe), grade 4 (life-threatening), grade 5 (death). In Part 1, ongoing monitoring of open-label safety data was conducted by the trial sponsor at regular intervals in addition to the routine safety surveillance activities, including but not limited to signal detection monitoring.

Data and Statistical Analyses

Data from Part 1 were to be included in the overall safety/tolerability summaries. However, Part 1 was designed for estimation without formal statistical testing, and there were no formal power considerations. The sample size enrolled (up to 10 per dose level) was designed to assess safety and inform the dose selection for Part 2 of the study.

Supporting Figure 1. Study Design: Open-Label Proof-of-Concept Phase for OHANA Trial. The figure depicts the design of Part 1 of the trial, including screening, eligibility, planned treatment, and key endpoints. BID, twice daily; sBA, serum bile acid; VLX, volixibat.

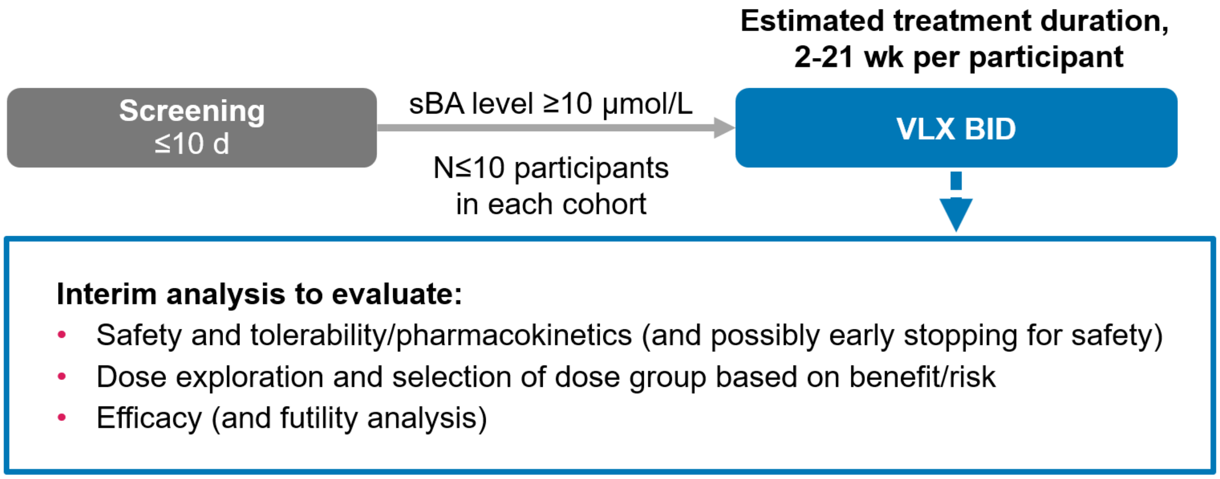

Supporting Table 1. Safety Outcomes

Participant	GI TEAEs reported	Number of events	Severity	Duration, d
1	Diarrhea	1	Grade 1	3
2	Diarrhea	3	Grade 2	2
			Grade 2	13
			Grade 3	2
3	Diarrhea	1	Grade 2	5
4	Diarrhea	2	Grade 2	18
			Grade 3	2

GI, gastrointestinal; TEAE, treatment-emergent adverse event.
